# Supplementary material for: A high-resolution mRNA expression time course of embryonic development in zebrafish
Source: eLife. 2017 Nov 16;6:e30860. doi: 10.7554/eLife.30860 (PMC5690287; doi:10.7554/eLife.30860)
Supplement: Supplementary file 6. [file elife-30860-supp6.zip › biolayout-clusters-files/Cluster094.html]

Cluster094


# Cluster094: Detail

### Go to ZFA detail

## GO

| GO ID | Description | Domain | Annotated | Expected | Observed | Adjusted p-value | Genes | Ensembl IDs |
| --- | --- | --- | --- | --- | --- | --- | --- | --- |
| GO:0042775 | mitochondrial ATP synthesis coupled elec... | biological\_process | 14 | 0.01 | 2 | 0.017 | ndufb9 cox8a | ENSDARG00000041314 ENSDARG00000095273 |
| GO:0005746 | mitochondrial respiratory chain | cellular\_component | 21 | 0.02 | 2 | 0.041 | ndufb9 cox8a | ENSDARG00000041314 ENSDARG00000095273 |
| GO:0004129 | cytochrome-c oxidase activity | molecular\_function | 22 | 0.02 | 2 | 0.041 | cox6b2 cox8a | ENSDARG00000037860 ENSDARG00000095273 |
